# Quasi-Static Pull-in: an Instability in Electrostatic Actuators

**DOI:** 10.1038/s41598-020-61534-w

**Published:** 2020-03-19

**Authors:** M. S. Al-Ghamdi, M. E. Khater, E. M. Abdel-Rahman, E. G. Nepomuceno

**Affiliations:** 1 National Center for Electronics and Photonics, King Abdulaziz City for Science and Technology (KACST), Riyadh, Saudi Arabia; 2 Mechanical Engineering, King Fahd University of Petroleum and Minerals (KFUPM), Dhahran, Saudi Arabia; 3 Department of Systems Design Engineering, University of Waterloo, Waterloo, ON Canada; 4 Department of Electrical Engineering, Federal University of Sao Joao del-Rei, Sao Joao del-Rei, MG Brazil

**Keywords:** Engineering, Nanoscience and technology, Physics

## Abstract

We identify a new instability in electrostatic actuators dubbed quasi-static pull-in. We report experimental evidence of the instability and study its characteristics in two types of micro actuators operating in ambient air. We found that the underlying mechanism is a fast-slow dynamic interaction between slowly-varying electrostatic excitation and fast resonator response that instigate large non-resonant oscillatory orbits and eventually disappears in a global Shilnikov bifurcation. Based on these findings, we formulate and present a new taxonomy of pull-in instabilities in electrostatic actuators.

## Introduction

Nonlinearities in electrostatic Micro-Electro-Mechanical Systems (MEMS) are a rich source of interesting dynamic phenomena. Sources of nonlinearity in electrostatic MEMS include the dependence of the electrostatic force on displacement, geometric and inertial nonlinearities, nonlinear damping mechanisms, and interactions with the substrate^1^. They result in static and dynamic bifurcations, multivaluedness, and chaos. These phenomena have been exploited to design high sensitivity sensors, large amplitude actuators, mechanical memory bits, and encryption keys^2–6^.

One of the most important nonlinear phenomena in electrostatic MEMS is the pull-in instability^7^ where the moving structure snaps to the actuation electrode. While significant efforts have been devoted to study this phenomenon, a consistent taxonomy of its different types and underlying mechanisms is yet to emerge. We posit that a classification system based on the ratio of frequency *f* of the excitation to the actuator’s fundamental natural frequency *f*_*n*_ can achieve that.

Static pull-in is measured using quasi-static ramp waveforms, (*f*/*f*_*n*_ → 0), which minimize inertial and damping effects. As the voltage between the actuator and an electrode increases monotonically, the stable equilibrium (node) and unstable equilibrium (saddle) coincide at a saddle-node bifurcation. Beyond this point, the actuator snaps to the electrode. Krylov and Maimon^8^ and Khater *et al*.^3^ utilized ramp waveforms with frequencies of *f* = 1 kHz and *f* = 1.8 Hz, respectively, to measure static pull-in. While a step or other waveforms may also be used, the common characteristic of static pull-in is transient (non-repeatable) dynamics.

A margin of stability exists around the saddle-node bifurcation. The size of this margin is proportional to the waveform rise time and the actuator quality factor^9^. A slow rise allows for accurate determination of the bifurcation point corresponding to static pull-in voltage. A fast rise instigates transients, thereby decreasing the effective static pull-in voltage. Many researchers have investigated the boundaries of this margin^8,10,11^.

Dynamic pull-in is instigated by resonant waveforms where the excitation frequency and one of the natural frequencies are integer multiples or submultiples of each other (*f*/*f*_*n*_ ≈ *p*∕*q*), such as the case for primary, superharmonic, and subharmonic resonances^12,13^. Dynamic pull-in occurs at lower RMS voltage than static pull-in because of the dynamic amplification available at resonance^14^. In low damping regimes, it occurs subsequent to a homoclinic tangle resulting in erosion of the safe motion basin^13,15,16^ when it reaches about 50% of the basin size^17^. It may also occur subsequent to a cyclic-fold bifurcation^13,17,18^. In high damping regimes, it occurs due to a homoclinic bifurcation as an orbit touches the stable manifold of the saddle^16^. Further, in all cases a margin of stability exists around the stable manifold of the saddle. Transients around a stable orbit that cause the actuator to wander beyond this margin also lead to dynamic pull-in^14^.

In this work, a new class of pull-in instabilities, dubbed quasi-static pull-in, is observed in orbital (repeatable) non-resonant dynamics where the excitation frequency is finite but much smaller than the fundamental natural frequency, *f*/*f*_1_ < < 1. It was observed in two actuators excited by biased AC voltage in ambient air. Table 1 summarizes the conditions and mechanisms of those instabilities. This paper reports on the identification of this instability and characterization of its underlying mechanism: a global Shilnikov bifurcation^19^.

**Table 1:** A taxonomy for pull-in instabilities.

Type	Frequency Ratio	Mechanism
Static	*f*/*f*_1_ → 0	Saddle-node bifurcation
Quasi-static	*f*/*f*_1_ < < 1	Shilnikov bifurcation
Dynamic	*f*/*f*_*n*_ ∝ *p*/*q*	(i) homoclinic bifurcation
		(ii) homoclinic tangle
		(iii) cyclic-fold bifurcation

Shilnikov bifurcation is characterized by the appearance of an orbit homoclinic to a saddle focus relevant to our case arises due to the presence of an unstable equilibrium (a saddle) characterized by the three eigenvalues closest to the imaginary axis having the form: (−*ρ* ± *iω*, *λ*) where *λ*, *ρ* > 0^19,20^. The orbit spirals towards the saddle then departs away from it in a large excursion along its unstable manifold. The ratio of the real part of the complex pair of eigenvalues to the real eigenvalue *δ* = *ρ*/*λ* is called the saddle index. When *δ* < 1 Shilnikov homoclinic orbits appear and Shilnikov chaos exists in their neighborhood^19,20^.

Shilnikov homoclinic orbits and Shilnikov chaos were previously observed in feedback controlled single mode laser^21^, intracavity multimode solid-state laser^22^, DC voltage excited plasma at a low gas pressure^23^, and fluid flows in concentric rotating cylinders^24^. In this paper, we report observation of Shilnikov orbits homoclinic to a saddle focus as well as Shilnikov chaos in electrostatic MEMS actuators undergoing quasi-static pull-in.

## Methods

**Figure 1:**
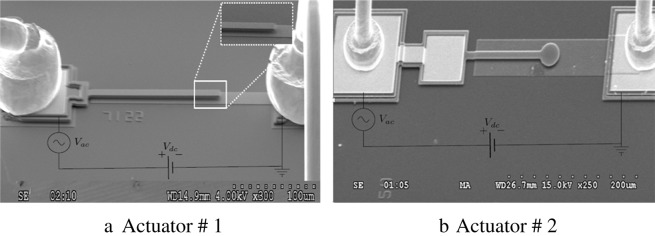
Scanning Electron Microscopy (SEM) pictures of the actuators (1) and (2) which consist of cantilever beam and cantilever beam attached with a plate at the end, respectively.

Experiments were conducted on two types of electrostatic MEMS actuators vibrating in their first out-of-plane bending mode. Actuator # 1, Fig. 1(a), is a microcantilever beam, whereas actuator # 2, Fig. 1(b), has a circular plate at the end of the beam. Substrate electrodes provide electrostatic actuation.

The actuators were fabricated using PolyMUMPs fabrication process^25^. The beams were fabricated in Poly 1 structural layer with dimensions of (175 × 10 × 2 μm^3^) for actuator # 1 and (115 × 10 × 2 μm^3^) for actuator # 2. Substrate electrodes were patterned in Poly0 layer under the full beam length. The capacitive gap was experimentally found to be *d* = 2 μm for both actuators. Two gold pads were patterned at the root of the beam and the end of the bottom electrode to apply potential difference to the actuator.

Experiments were conducted in atmospheric pressure while the actuators were placed inside a metal enclosure to protect against stray magnetic fields. A function generator was used to supply the actuation waveform. A laser-Doppler vibrometer^26^ was utilized to measure the actuator tip velocity and displacement. The measurements were digitized using a digital oscilloscope.

## Results

**Figure 2:**
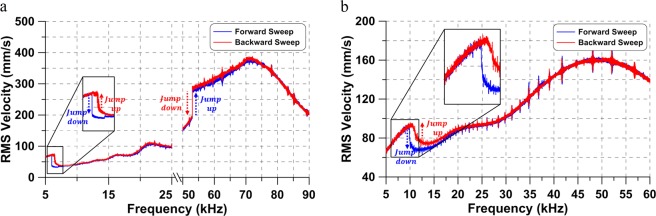
The frequency-response curves of (**a**) actuator #1 under the excitation voltage *V*_*dc*_ = *V*_*ac*_ = 7.725 V and (**b**) actuator # 2 under the excitation voltage *V*_*dc*_ = *V*_*ac*_ = 7.125 V. Forward and backward frequency-sweeps are colored in blue and red, respectively.

The frequency-response curves of the tip velocity, shown in Fig. 2, were obtained under constant voltage waveforms to characterize the actuators response over a wide frequency range. Each curve is composed of a forward and a backward frequency sweep. The voltage waveform and frequency range were *V*_*dc*_ = *V*_*ac*_ = 7.725 V and *f* = [5–90] kHz for actuator # 1 and *V*_*dc*_ = *V*_*ac*_ = 7.125 V and *f* = [5–60] kHz for actuator # 2. The slew rate was set to 2.5 kHz/s to minimize transient effects. Data was collected in time windows of 0.4 s at a sampling frequency of *f*_*s*_ = 313 kHz. The time-domain data was post-processed to obtain the RMS velocity of the tip over a time window of 20 excitation periods (*T*) and assigned to the frequency value at the window mid-point.

For both actuators, no motions were observed for excitation frequencies lower than 5 kHz with the actuator going into and remaining in contact with the substrate throughout the excitation cycle. Periodic and aperiodic motions appeared for excitation frequencies *f* ≥ 5 kHz. The positive slope lines observed in the frequency ranges *f* = [5.0–6.490] kHz and *f* = [53.0–70.0] kHz for actuator # 1 and *f* = [5.0–10.627] kHz for actuator # 2 are evidence of tapping-mode oscillations where the actuator tip comes into regular contact with the substrate. In these ranges, the limiting effect of the substrate maintains the tip displacement almost constant at a value close to the gap distance *d* = 2 μm. As a result, the velocity frequency-response varies almost linearly with frequency.

The tapping-mode oscillations observed at low-frequency are large even though they occur faraway from primary resonance *f*/*f*_1_ < < 1 and its superharmonics. In fact, while actuator # 1 also experiences similar sized tapping mode oscillations and dynamic pull-in at primary (*f* ≈ *f*_1_) resonance, actuator #2 does not experience the large orbits, leading to tapping or pull-in, except in that low frequency range. Further, the low-frequency tapping-mode orbits jump-down during forward frequency sweeps to a branch of smaller freely oscillating orbits, whereas a jump-up occurs from that branch to the tapping-mode branch during backward sweeps. This behavior is the reverse of that observed in the hysteric region located in the vicinity of primary resonance where the jump-up occurs during forward sweeps and the jump-down during backward sweeps. These distinctions raise questions about the nature of those large oscillations away from resonance and the nature of the pull-in instability they trigger.

### Quasi-Static Pull-in - Actuator # 1

**Figure 3:**
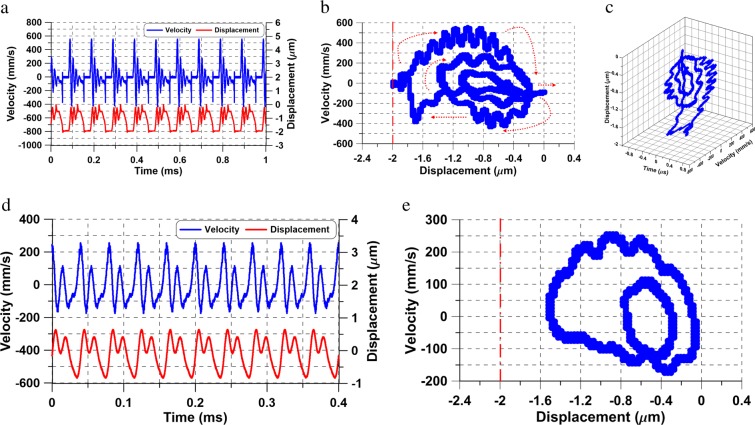
The time-histories of actuator # 1 tip velocity (blue line) and displacement (red line) under the voltage waveform *V*_*ac*_ = *V*_*dc*_ = 6.863 V and a signal frequency of (**a**) *f* = 10 kHz and (**d**) *f* = 25 kHz. The corresponding 2-D phase portraits (**b**) at *f* = 10 kHz, and (**e**) *f* = 25 kHz. (**c**) The 3-D phase portrait reconstructed from the time-histories shown in (**a**).

We compare this actuator’s response under the same voltage waveform *V*_*ac*_ = *V*_*dc*_ = 6.863 V at two excitation frequencies *f* = 10 and 25 kHz. The velocity and displacement time-histories of the actuator tip are shown in Fig. 3(a,d). Planar projections of the corresponding phase-portraits are shown in Fig. 3(b,e) while a 3-D phase-portrait of the orbit at *f* = 10 kHz is shown in Fig. 3(c).

The response at *f* = 10 kHz displays the large oscillations mentioned above unlike the case at *f* = 25 kHz. The time-history of the displacement and velocity in Fig. 3(a) shows that as the voltage varies, the actuator tip is pulled-in periodically coming into contact with the substrate where the displacement is held constant for a period of time before pulling off and settling down close to the equilibrium position. The phase-portrait at *f* = 10 kHz reveals a Shilnikov orbit homoclinic to a saddle-focus, Fig. 3(c). The orbit is composed of a flow along the unstable manifold away from the saddle, captured by impact and rebound on the substrate, reinjected into the vicinity of the stable focus, where it settles down, before the excitation waveform displaces it once again towards the saddle^27^. The process of pulling-in corresponds to the flow along the unstable manifold of the saddle while settling down around the stable equilibrium corresponds to the stable focus. Flow again towards the saddle occurs due to the cyclic rise in instantaneous voltage *V*(*t*) as it approaches the static pull-in voltage *V*_*Ps*_ ≈ 15.60 V.

We note that concepts of static pull-in do not apply to this process. The RMS of the voltage waveform *V*_RMS_ = 11.716 V is significantly less than *V*_*Ps*_. Further, the response is periodic and free of transients while static pull-in is fundamentally a transient phenomenon. Beyond the bifurcation point at the peak of the tapping branch, the actuator jumps down to a stable forced response orbits as shown in Fig. 3(d,e) at *f* = 25 kHz with the orbit size along the displacement-axis shrinking from 2 μm at *f* = 10 kHz to 1.120 μm at *f* = 25 kHz.

**Figure 4:**
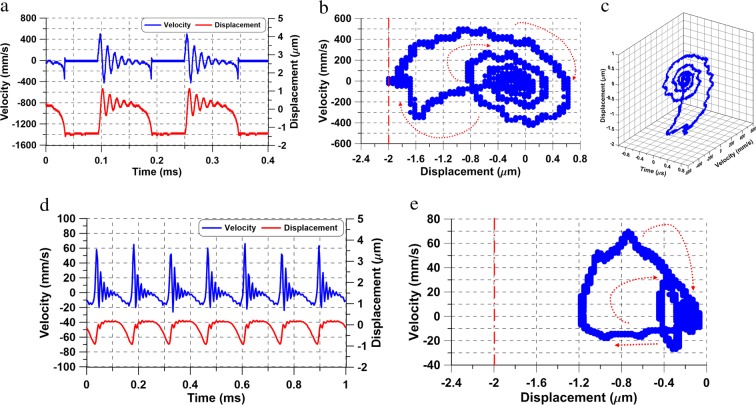
The time-histories of actuator # 1 tip velocity (blue line) and displacement (red line) under the voltage waveform *V*_*ac*_ = *V*_*dc*_ = 7.725 V and a signal frequency of (**a**) *f* = 6.40 kHz and (**d**) *f* = 7.0 kHz. The corresponding 2-D phase portraits (**b**) at *f* = 6.40 kHz, and (**e**) *f* = 7.0 kHz. (**c**) The 3-D phase portrait reconstructed from the time-histories shown in (**a**).

Increasing the excitation voltage to *V*_*ac*_ = *V*_*dc*_ = 7.725 V reduces the frequency domain where Shilnikov orbits and large oscillations occur. Experimentally measured velocity and displacement time-histories and the corresponding phase portraits at excitation frequencies of *f* = 6.40, and 7.0 kHz are shown in Fig. 4 for time spans of 2.560, and 7 excitation periods, respectively.

While a similar behavior to that described above is observed here, the bifurcation point is lactated at the peak of the tapping branch *f* = 6.49 kHz. Similarly, the orbit size shrinks from a displacement of 2.5 μm at *f* = 6.4 kHz to 1.1 μm at *f* = 7.0 kHz. While oscillations typical of a stable focus can be observed in Fig. 4(e), they result from the actuator rebounding from its maximum deflection point as can be seen in the corresponding time-history, Fig. 4(d). The complexity of the orbit is merely a reflection of the interaction between the slow time-scale of forcing and the fast time-scale of the actuator’s fundamental mode. No tapping or flow along the unstable manifold is observed in Fig. 4(d,e). The fast-slow dynamics in this region result in two distinct trains of peaks in frequency-domain, corresponding to the forcing frequency and to the fundamental natural frequency of the actuator. For this waveform, we observed fast-slow dynamic interactions in the region extending from the bifurcation point (*f* = 6.49 kHz) until approaching the superharmonic resonance of order-three (*f* ≈ 25 kHz).

### Quasi-Static Pull-in - Actuator # 2

**Figure 5:**
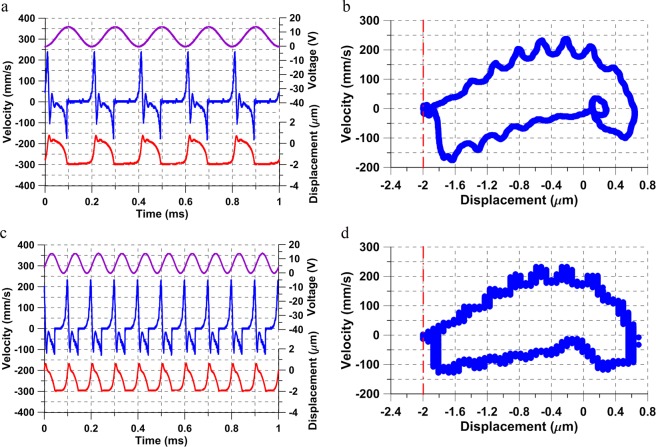
The voltage waveform (magenta line) and time-histories of actuator # 2 tip velocity (blue line), and displacement (red line) under the voltage waveform *V*_*ac*_ = *V*_*dc*_ = 6.750 V and a signal frequency of (**a**) *f* = 5.0 kHz and (**c**) *f* = 10 kHz. The corresponding phase portraits at (**b**) *f* = 5.0 kHz and (**d**) *f* = 10 kHz.

We compare this actuator’s response under the voltage waveform *V*_*ac*_ = *V*_*dc*_ = 6.750 V at two excitation frequencies *f* = 5 and 10 kHz. The tip displacement and velocity time-histories and the corresponding phase-portraits are shown in Fig. 5. Shilnikov orbits homoclinic to a saddle focus were observed in both cases, Fig. 5(b,d). The stable focus oscillations occur over a shorter settling time because of the actuator’s lower quality factor (*Q* = 2.1). Comparison of the voltage waveform to the displacement time-history, Fig. 5(a,c), shows that the actuator pulls-in as the instantaneous voltage crosses the value of static pull-in, (*V*_*Ps*_ ≈ 12.15 V). Subsequently, it maintains contact with the substrate until the instantaneous voltage drops below pull-off voltage.

**Figure 6:**
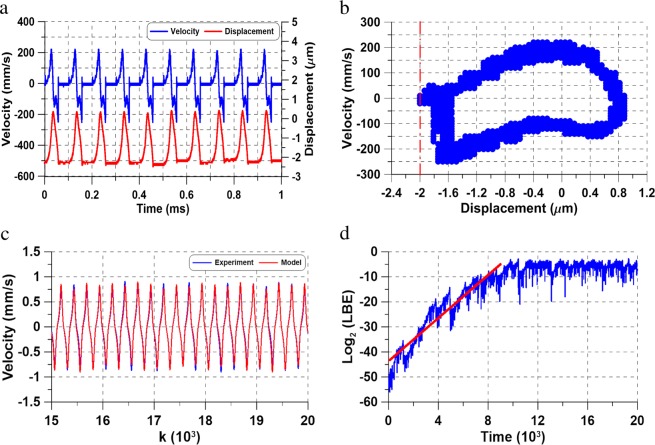
(**a**) The time-histories of actuator # 2 tip velocity (blue line) and displacement (red line) under the voltage waveform *V*_*ac*_ = *V*_*dc*_ = 7.650 V and an excitation frequency of *f* = 10 kHz, (**b**) the corresponding phase portraits, (**c**) comparison of measured (blue line) and simulated (red line) velocity time-histories and (**d**) computation of the largest positive Lyapunov exponent (LLE).

The voltage waveform was increased to *V*_*ac*_ = *V*_*dc*_ = 7.650 V for the excitation frequency *f* = 10 kHz, Fig. 6, which resulted in Shilnikov chaos. The velocity and displacement time-histories of the chaotic tapping oscillations are shown in Fig. 6(a). The corresponding phase portrait, Fig. 6(b), shows a banded chaotic attractor. This chaotic attractor was found to extend over the frequency [10–18] kHz during a forward sweep.

To estimate the Largest Lyapunov Exponent (LLE), a nonlinear autoregressive moving average model (NARMAX) was first identified from the velocity time-history. A comparison of the model predicted (red lines) and measured (blue lines) velocity over twenty excitation periods is shown in Fig. 6(c) to confirm the model validity. The logarithmic lower bound error (LBE) between two equivalent extensions of the model^28^ is shown in Fig. 6(d). The LLE was estimated from the slope of the line (*LBE* = 0.004*t* − 43.67) approximating the initial growth of LBE as 2 × 10^−6^ bit/s. The positive LLE establishes that the underlying attractor is chaotic.

## Discussion and Conclusions

We identified a new type of pull-in instabilities in electrostatic actuators dubbed quasi-static pull-in. It occurs under periodic excitation, as opposed to the case for static pull-in, in a non-resonant frequency range much lower than the fundamental natural frequency *f*/*f*_1_ < < 1, as opposed to the case for dynamic pull-in. The instability was replicated and verified in two independent MEMS electrostatic actuators oscillating in ambient air.

The transition from static pull-in to quasi-static pull-in occurred in both cases at 5 kHz and corresponded to the beam tip contact with the substrate transitioning from area-contact to line-contact. In all cases, the peak instantaneous voltage *V*(*t*)_*Max*_ was close to but below static pull-in voltage, thereby allowing transients to cause pull-in. To analyze this transition, we write the force balance driving the actuator’s upward acceleration $\ddot{x}$ as:

1 $$m\ddot{x}={F}_{rs}+{F}_{rb}-{F}_{e}-{F}_{s}$$

where *m* is the effective mass, *F*_*rs*_ restoring force, *F*_*rb*_ rebound force recovered from momentum before impact, *F*_*e*_ electrostatic force, and *F*_*s*_ net surface stiction force. We further note that *F*_*rs*_ and *F*_*s*_ are functions of the contact condition rather than the voltage waveform. Both forces are larger when area-contact develops than they are when line-contact prevails^29^. On the other hand, *F*_*rb*_ increases with the excitation frequency as the velocity of the actuator increases and *F*_*e*_ is proportional to the square of the waveform *V*^2^(*t*) and larger in area-contact than line-contact.

At lower frequencies, the rebound force *F*_*rb*_ is low and the electrostatic force *F*_*e*_ drops slowly towards zero, thereby allowing the actuator to evolve into area contact and resulting in a larger surface contact force *F*_*s*_. The force balance in this case *F*_*rs*_ + *F*_*rb*_ − *F*_*e*_ − *F*_*s*_ < 0 prevents pull-off, and therefore motion. Beyond 5 kHz, the rebound force *F*_*rb*_ is larger and the electrostatic force drops faster towards zero preventing area-contact and limiting the surface contact force to smaller values. The resulting force balance *F*_*rs*_ + *F*_*rb*_ − *F*_*e*_ − *F*_*s*_ > 0 favors pull-off.

Therein lies a fundamental difference between static pull-in and quasi-static pull-in: transient (non-repeatable) dynamics underlie the first whereas orbital dynamics underlie the second. Quasi-static pull-in requires oscillatory forcing at a frequency high enough to initiate pull-off. A necessary condition for that are low surface stiction forces.

Quasi-static pull-in is driven by a fast-slow dynamic interaction between slowly varying electrostatic excitation and fast response of the actuator’s fundamental mode. It is triggered by excitation waveforms where the instantaneous voltage approaches or exceeds static pull-in voltage *V*_*Ps*_ resulting in the appearance of Shilnikov orbits homoclinic to a saddle-focus. Those orbits are characterized by large tapping-mode oscillations where the actuator periodically goes to pull-in through the saddle (unstable equilibrium), pulls-off and settles around the stable equilibrium, before drifting again towards the saddle to repeat the cycle. The actuator’s quality factor determines the order of the homoclinic orbit (settling time around the static equilibrium)^30^. The homoclinic orbit observed in actuator # 1 (*Q* = 5.4) was of order 5, whereas those observed in actuator # 2 (*Q* = 2.1) were of order 1.

In forward frequency-sweeps, Shilnikov homoclinic orbits appear beyond static pull-in, persist along a branch of tapping-mode oscillations, and evolve into Shilnikov chaos. As the excitation frequency increases, the impact speed increases, thereby increasing impact-induced damping. Elevated damping reduces the real part of the saddle’s leading pair of complex eigenvalues *ρ*, therefore violating the condition for Shilnikov homoclinic orbits: *δ* = *ρ*/*λ* < 1. At that point, the branch terminates with the response falling down to a coexisting branch of stable freely oscillating periodic orbits. A Shilnikov bifurcation demarcates the end of the free oscillations branch where the response jumps-up to the tapping-mode branch during backward frequency-sweeps. A hysteretic region exists between these jumps, bounded at the lower-end by the jump-down in forward-sweeps and at the upper end by the jump-up in backward-sweeps. This is another characteristic of quasi-static pull-in in contrast to the hysteric region associated with dynamic pull-in where the jump-down occurs in backward-sweeps and the jump-up in forward-sweeps.
